# N-terminal truncated phospholipase A1 accessory protein PlaS from *Serratia marcescens* alleviates inhibitory on host cell growth and enhances PlaA1 enzymatic activity

**DOI:** 10.1186/s40643-024-00777-1

**Published:** 2024-06-25

**Authors:** Mengkai Hu, Jun Liu, Yufei Gan, Hao Zhu, Rumeng Han, Kun Liu, Yan Liu, Ming Zhao, Xiangfei Li, Zhenglian Xue

**Affiliations:** Engineering Laboratory for Industrial Microbiology Molecular Beeding of Anhui Province, College of Biologic & Food Engineering, Anhui Polytechnic University, 8 Middle Beijing Road, Wuhu, 241000 China

**Keywords:** Phospholipase A1, PlaS, N-terminal truncation, Protein-protein interaction, Enzymatic activity

## Abstract

Phospholipase A1 (PLA1) is a kind of specific phospholipid hydrolase widely used in food, medical, textile. However, limitations in its expression and enzymatic activity have prompted the investigation of the phospholipase-assisting protein PlaS. In this study, we elucidate the role of PlaS in enhancing the expression and activity of PlaA1 through N-terminal truncation. Our research demonstrates that truncating the N-terminal region of PlaS effectively overcomes its inhibitory effect on host cells, resulting in improved cell growth and increased protein solubility of the protein. The yeast two-hybrid assay confirms the interaction between PlaA1 and N-terminal truncated PlaS (∆N27 PlaS), highlighting their binding capabilities. Furthermore, in vitro studies using Biacore analysis reveal a concentration-dependent and specific binding between PlaA1 and ∆N27 PlaS, exhibiting high affinity. Molecular docking analysis provides insights into the hydrogen bond interactions between ∆N27 PlaS and PlaA1, identifying key amino acid residues crucial for their binding. Finally, the enzyme activity of PLA1 was boost to 8.4 U/mL by orthogonal test. Study significantly contributes to the understanding of the interaction mechanism between PlaS and PlaA1, offering potential strategies for enhancing PlaA1 activity through protein engineering approaches.

**Supplementary Information:**

The online version contains supplementary material available at 10.1186/s40643-024-00777-1.

## Introduction

Phospholipase A1 (PlaA1) is an important enzyme found in various organisms, including bacteria, fungi, and animals (Zhang et al. 2021; Park et al. 2020). As a phospholipase, PlaA1 catalyzes the hydrolysis of phospholipid molecules on cell membranes, leading to the generation of lyso-phospholipids and free fatty acids, playing crucial biological roles (Yang et al. 2019a, b; Aloulou et al. 2018). PlaA1 plays essential roles in multiple biological processes, including cell signaling, inflam-matory responses, and immune reactions. However, previous studies have shown that the activity of phospholipase A1 is restricted by various factors, including enzyme ex-pression levels and enzymatic activity (Yang et al. 2019a, b; Lim et al. 2016). To address these limitations, researchers have discovered a protein called phospholipase A1 Accessory Protein (PlaS). PlaS is a protein that coexists with PlaA1, interacts with it, and influences its expression and activity.

PlaS has been widely identified in various organisms, particularly in certain bacteria (Song et al. 1999). Research conducted within our laboratory has unveiled both the structural and functional attributes of PlaS derived from *Serratia marcescens* (Li et al. 2023). Through analysis of its signal peptide sequence, we have uncovered the existence of an N-terminal signal peptide in PlaS, which is likely instrumental in its localization and transportation mechanisms. Additionally, investigations have pinpointed the presence of a hydrophobic domain and a transmembrane region at PlaS’s N-terminal, hinting at potential implications for its interaction with PlaA1 and its related functions. While initial insights into the function and mechanisms of PlaS have been gained, numerous questions still remain unanswered. For instance, how does PlaS specifically interact with PlaA1? How does PlaS regulate the expression and activity of PlaA1? Addressing these questions will provide a deeper understanding of the role of PlaS in PlaA1-mediated processes and pave the way for potential therapeutic applications targeting this interaction.

In recent years, N-terminal truncation technology has emerged as a cutting-edge approach with wide-ranging applications in various fields of research (Liu et al. 2019; Li et al. 2018a, b; Zhou et al. 2022). This technique involves removing a portion of the N-terminal domain of a protein, resulting in modified protein variants with altered properties and functionalities. N-terminal truncation technology has revolutionized protein engineering (Rosen and Francis 2017), drug discovery (Liu et al. 2020), and biotechnological applications (Deng et al. 2020), offering new avenues for exploring protein structure-function relationships and manipulating protein behavior. N-terminal truncation technology has found extensive use in the study of protein-protein interactions (Sista Kameshwar and Qin 2018; Dunham et al. 2009), protein folding (Ahn et al. 2022), and enzymatic activities (Lu et al. 2016; McGlinchey et al. 2021). By selectively removing specific amino acid residues from the N-terminal region, researchers can investigate the role of this domain in protein stability, localization, and interaction with other molecules (Dunham et al. 2009; Ahn et al. 2022; Lu et al. 2016). Furthermore, this technology has proven particularly valuable in enhancing the catalytic efficiency, substrate specificity, and thermostability of enzymes (Lu et al. 2016). Therefore, the altered conformation induced by N-terminal optimization can influence the catalytic efficiency and substrate specificity of PlaA1, ultimately enhancing its enzymatic performance. These findings highlight the potential of N-terminal optimization as a valuable tool for improving the functional properties of PlaA1 and PlaS.

In this study, we aimed to investigate the impact of N-terminal truncation on the function of PlaS and its interaction with PlaA1. N-terminal truncation of PlaS was levered to alleviate its inhibitory effect on host cells, leading to enhanced PlaA1 activity. Our experimental design involved truncating the N-terminal region of PlaS and evaluating its effects on cell growth, protein expression, and the interaction with PlaA1 using the yeast two-hybrid assay. Additionally, we employed Biacore analysis to assess the binding kinetics between PlaA1 and truncated PlaS. Our results demonstrate that N-terminal truncation of PlaS effectively overcomes its inhibitory effect on host cells, allowing improved cell growth and increased protein expression. The yeast two-hybrid assay confirms the interaction between PlaA1 and ∆N27 PlaS, highlighting their binding capabilities. Biacore analysis reveals a concentration-dependent and specific binding between PlaA1 and ∆N27 PlaS, with high affinity. Furthermore, molecular docking analysis provides insights into the hydrogen bond interactions between ∆N27 PlaS and PlaA1, identifying key amino acid residues crucial for their binding.

## Materials and methods

### Strains, plasmids, and growth conditions

The strains, plasmids and primers used in this study were listed in Table S1 and Table S2, respectively. *Escherichia coli* BL21(DE3) and *E. coli* DH5α were deposited in the laboratory. *E. coli* DH5α and *E. coli* BL21 cells were used as hosts for cloning and expression of target protein, respectively. The plasmids pGBKT7, pGADT7, pGBKT7-53, pGBKT7-lam, pGADT7-T and Y2HGold yeast competent cells were provided by Wuhan Genecreate Biotechnology Co., Ltd. The plasmid vector pET28a was used to express target proteins. *E. coli* BL21 and *E. coli* DH5α cells were grown in LB medium at 37 °C, Y2HGold was grown in YPDA medium. SD/-Trp (SDO) medium (g/L): 6.7 g YNB, 0.74 g SD/-Trp, 20 g glucose, pH 5.8. SD/-Trp/X (SDO/X) medium (g/L): 6.7 g YNB, 0.74 g SD/-Trp, 20 g glucose, 200 µL X-a-Gal (200 mg/mL), pH 5.8. SD/-Trp/X/A (SDO/X/AbA) medium (g/L): 6.7 g YNB, 0.74 g SD/-Trp, 20 g glucose, 200 µL X-a-Gal (200 mg/mL), 200 µL Aba (5 mg/mL), pH 5.8. SD/-Leu/-Trp (DDO) medium (g/L): 6.7 g YNB, 0.67 g SD/-Leu/-Trp, 20 g glucose, pH 5.8. SD/-Leu/-Trp/X (DDO/X) medium (g/L): 6.7 g YNB, 0.67 g SD/-Trp, 20 g glucose, 200 µL X-a-Gal (200 mg/mL), pH 5.8. SD/-Leu/-Trp/X (DDO/X) medium (g/L): 6.7 g YNB, 0.74 g SD/-Trp, 20 g glucose, 200 µL X-a-Gal (200 mg/mL), pH 5.8. SD/-Leu/-Trp/-His (TDO) medium (g/L): 6.7 g YNB, 0.62 g SD/-Leu/-Trp/-His, 20 g glucose, pH 5.8. SD/-Leu/-Trp/-His/-Ade (QDO) medium (g/L): 6.7 g YNB, 0.60 g SD/-Leu/-Trp/-His/-Ade, 20 g glucose, pH 5.8. SD/-Leu/-Trp/-His/-Ade/Aba/X (QDO/X/A) medium (g/L): 6.7 g YNB, 0.60 g SD/-Leu/-Trp/-His/-Ade, 200 µL X-a-Gal (200 mg/mL), 40 µL Aba (5 mg/mL), 20 g glucose, pH 5.8. All the above solid media should be supplemented with 20 g/L agar powder.

### Construction of plasmids and the recombinant strains

The target fragments plaS and ∆N27 PlaS were amplified with the genome of *S. marcescens* as a template and the primer pair plaS F/plaS R and ∆N27 PlaS F/∆N27 PlaS R, respectively. ClonExpress II One Step Cloning Kit (Vazyme Biotech Co., Ltd., Nanjing, China) (Li et al. 2021) was used to connect the obtained target DNA fragments and the linearized vector pET28a that had been digested and purified with *Xho*I and *Hind*III to construct recombinant plasmids pET28a-PlaS and pET28a-∆N27 PlaS, respectively. The plasmids pET28a-PlaA1-PlaS and pET28a-PlaA1-∆N27 PlaS were constructed in the same way. The two plasmids were constructed using the pET28a-PlaA1 plasmid as the starting plasmid, then the fragments of plas and ∆N27 PlaS were inserted into the BamHI and EcoRI sites of the vector pET28a-PlaA1, respectively, where PlaA1 was inserted into the *Xho*I and *Hin*dIII restriction sites of the plasmid. The plasmids pGBKT7 and pGADT7 was digested and purified with *Eco*RI and *Bam*HI, and *Xho*I and *Hind*III to obtain the linearized vector, respectively. Then, ClonExpress II One Step Cloning Kit (Vazyme Biotech Co., Ltd., Nanjing, China) was used to connect the target DNA fragments PlaA1, PlaS and ∆N27 PlaS with the linearized vector pGBKT7 and pGADT7. The recombinant plasmids pGBKT7-PlaA1, pGADT7-PlaS and pGADT7-∆N27 PlaS was transformed into Y2HGold by electro transformation (López-Jimenez et al. 2021). All the recombinant plasmids constructed were transformed into *E. coli* and identified by colony PCR and sequencing at the be-ginning. The method for the yeast two-hybrid assay was performed as described (Bacon et al. 2021; Wang et al. 2021).

### Molecular docking

SWISS-MODEL (https://swissmodel.expasy.org/interactive) was used to generate three-dimensional protein models through homology modeling for molecular docking of PlaS and PlaA1. The docking calculation of protein is obtained by ZDOCK in Dis-covery Studio (Yuan et al. 2023). In simple terms, using Discovery Studio, open the PDB file of the target molecule, and define PlaA1 as the receptor molecule and ∆N27 PlaS as the lig-and molecule for molecular docking. Then, utilize the “Macromolecules | Dock and Analyze Protein Complexes | ZDOCK” tool in Discovery Studio for protein-protein molecular docking. The fold recognition method allows the search for folders that match PlaA1 and ∆N27 PlaS to a high degree of confidence for molecular docking. The interactions between PlaA1 and ∆N27 PlaS were analyzed by Discovery Studio.

### Expression and purification of target proteins

Recombinant strains BL21/pET28a-PlaS and its mutants were cultured in LB me-dium to an OD_600_ = 0.5 at 37 °C, 180 rpm. Then 0.5 mM isopropyl β-D-1-Thiogalactopyranoside (IPTG) was added and cells was transferred to 16 °C, 180 rpm to induce protein expression. After induction of expression for 10–12 h, Cells were collected by centrifugation at 8000 rpm for 10 min at 4 °C, re-suspended and washed twice with Tris-HCl buffer (50 mM Tris-HCl, pH 7.4). Then the resuspended cells were treated with ultrasonic cell fragmentation apparatus for 10 min. The supernatants were centrifuged at 4 °C, 12,000 rpm for 30 min to collect the crude enzyme solution. Ni^2+^-affnity chromatography and an AKTA purifer system (GE Healthcare, Sweden) were used to purify the crude enzyme. The obtained crude enzyme solution and purified enzyme could be used for SDS-PAGE analysis (Li et al. 2018a, b). To determine the intracellular and extracellular enzyme activities of PlaA1-PlaS and PlaA1-∆N27 PlaS, cells were cultured using the same aforementioned method. Protein expression was induced, fol-lowed by centrifugation to collect the cell-free supernatant for extracellular enzyme activity measurement. The cultured cells were then harvested by centrifugation, and the obtained cell pellets were subjected to ultrasonic cell fragmentation for 10 min. The resulting supernatant was collected by centrifugation at 4 °C, 12,000 rpm for 30 min and used as the crude enzyme solution for intracellular enzyme activity measurement.

### Enzyme activity determination of PlaA1

PlaA1 enzyme activity is defined as the amount of enzyme required to produce 1 micromolar (µmol) free fatty acid per minute by hydrolysis of lecithin at 45 °C as one enzyme activity unit (U). Enzymatic specific activity was defined as the amount of phospholipase A1 per mg of protein. Protein content was determined using the Bradford quantification method, and a protein standard curve was plotted. The produced fatty acid was determined by using commercial free fatty acid assay kit (Nanjing Jiancheng Bioengineering Institute, Nanjing, China) followed by the manufacture’s instruction. The standard curve of free fatty acid was established at 546 nm by using a microplate reader to calculate the amount of the produced free fatty acid. The absorbance value was measured at 595 nm by reacting the extracellular enzyme solution with Brilliant Blue G-250 solution, and the protein content was calculated from the protein standard curve. The specific enzyme activity was then calculated based on the enzyme activity size. The detailed steps are as follows: First, 4 ml of 8% lecithin is mixed with 5 ml of pH 7.0 100mM phosphate buffer (containing CaCl2 with a final concentration of 20 μm) at 45℃ for 5 min, and then 1 mL crude enzyme solution was added to react at 45 ℃ for 15 min. Finally, 6 ml 95% ethanol was added to terminate the reaction and titrated with 0.05 M NaOH until the pH value was 10.3. The control group was added 6 ml 95% ethanol followed by crude enzyme solution.

### Studies on enzymatic properties

To determine the optimal temperature and optimal pH of PlaA1 and mutants, other enzymatic reaction conditions were kept constant according to the above methods. The PlaA1 activity of strain BL21/pET28a-PlaA1-PlaS was defined as 100% under optimal conditions, and the relative enzyme activity of the other samples was calculated.

### Kinetic parameter

The enzymatic specific activity was measured by reacting lecithin as the substrate with a unit volume of enzyme solution for 15 min at pH = 6 and 45 °C. The lecithin concentrations were as follows: 0.02, 0.04, 0.06, 0.08 and 0.1 mg/mL, respectively. The reaction rate [V] was the ratio of enzymatic specific activity to the reaction time, and the inverse of the reaction rate and substrate concentration [S] was used as the coordinate to calculate the K_m_ and V_max_ value respectively according to the Lineweaver-Burk plot method (Figure S3).

### Spot board verification

Single colonies were selected from the experimental group, negative group and positive group on agar plate DDO, and inoculated into 5mL YPDA liquid medium, cultured at 250 rpm at 30 ℃ for 16 h, respectively. Then 300 µL bacterial solution was taken into 50 mL YPDA and cultured at 250 rpm at 30 ℃ for 8–12 h to OD600 value of 0.5. Finally, 10 µL bacterial solution was taken and coated on agar plate DDO, TDO 20mM 3-AT and QDO, and cultured in 30 ℃ incubator for 3 days to observe the growth of plaque.

### Optimization of phospholipase A1 activity

Orthogonal test was used to optimize the phospholipase A1 enzyme activity of co-expressing strain BL21/pET28a-PlaA1-∆N27 PlaS, three factors (Initial induced cell density, induced time, IPTG concentration) and three levels of L_9_(3^4^) were selected, as detailed in Table S3, and ANOVA analysis was conducted in Table S4.

### Bioinformatics analysis

The amino acid sequence of PlaS was analyzed using bioinformatics tools. Signal peptide analysis was performed using the SignalP server (http://www.cbs.dtu.dk/services/SignalP/), hydrophobic structure analysis using the ProtScale tool (https://web.expasy.org/protscale/), and transmembrane region analysis using the TMHMM server (http://www.cbs.dtu.dk/services/TMHMM/).

## Results and discussion

### N-terminal truncation promotes cell growth and PlaS protein expression in *E. Coli*

**Fig. 1:**
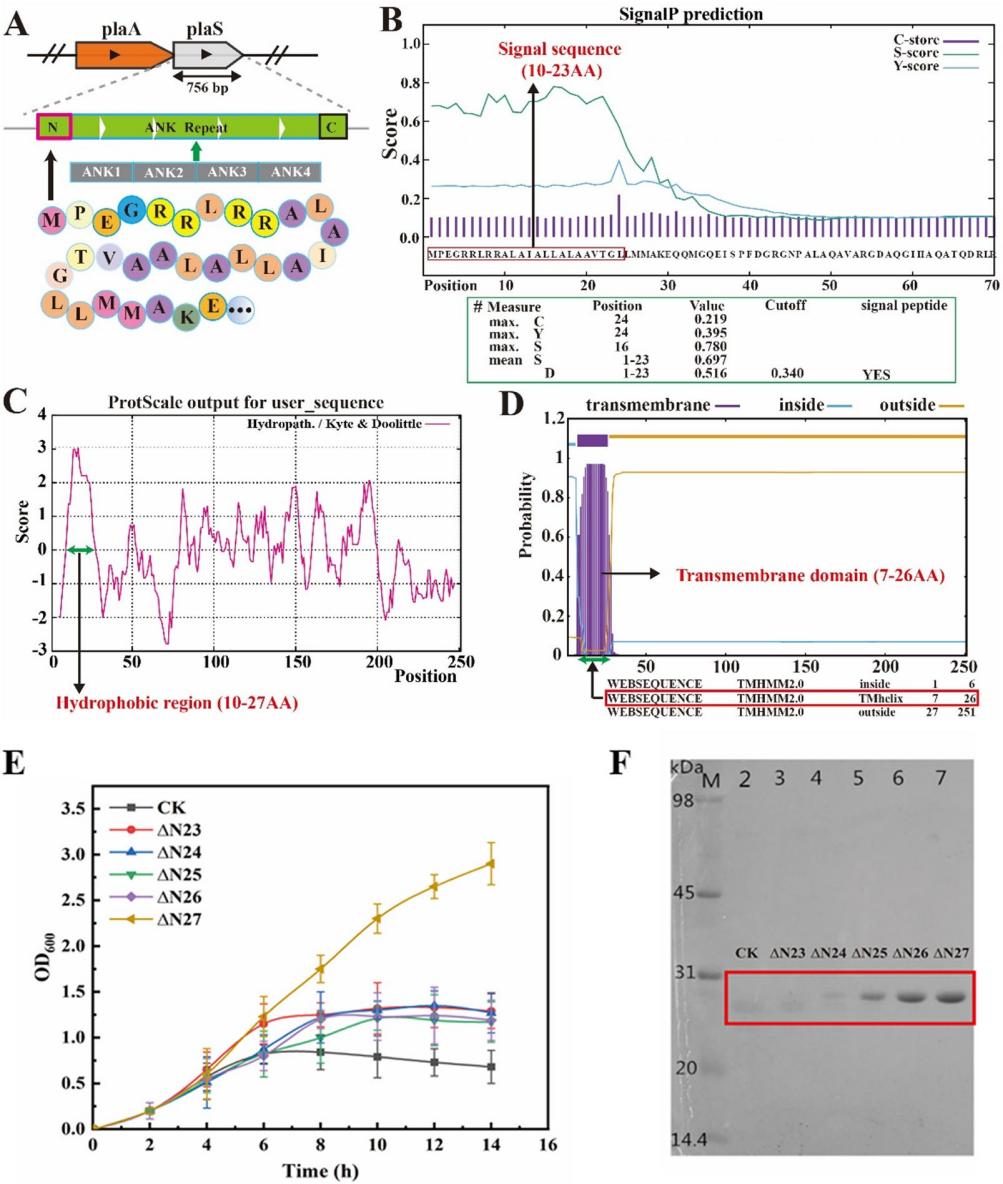
Auxiliary intracellular PlaS amino acid information analysis and N-terminal truncation of PlaS Protein. (**A**) Structure diagram of PlaS. (**B**) Signal peptide analysis. (**C**) Hydrophobic interaction analysis. (**D**) Transmembrane region analysis. (**E**) Growth curves of different recombinant strains after IPTG induction, showing the enhanced growth of N-terminal non-truncated PlaS mutants compared to the control strain. (**F**) SDS-PAGE analysis of PlaS protein with different truncation lengths, demonstrating increased solubility with N-terminal truncation

Previous laboratory research has identified a downstream gene coding sequence in the phospholipase A1 (PlaA1) gene from *Serratia marcescens* (Fu et al. 2008). This coding se-quence enhances the expression activity of PlaA1 and is named PlaS, the phospho-lipase-assisting protein (Fig. 1A). Analysis of PlaS using online tools revealed that the first 23 amino acids at the N-terminal region constitute the signal peptide domain, amino acids 7–26 form the transmembrane domain, and amino acids 10–27 comprise the hydrophobic region (Fig. 1B-D).

Studies have shown that the presence of exogenous signal peptides during pro-karyotic expression can affect proper protein expression (Freudl 2018; Chen et al. 2023; Owji et al. 2018), while removal of the hydrophobic region can effectively promote high-activity protein expression (Rego et al. 2021; Dhami et al. 2022). Previous studies found that expressing PlaS in *E. coli* BL21 significantly inhibited the growth of the host strain, resulting in very low protein expression levels and hindered purification of PlaS protein. Therefore, to eliminate the inhibitory effect of PlaS on the *E. coli* host and enhance high expression, we performed N-terminal truncation of PlaS by removing amino acids 23–27. Recombinant strains ∆N23, ∆N24, ∆N25, ∆N26, and ∆N27 were constructed using the expression vector pET28a in *E. coli* BL21. Measurement of cell growth in different recombinant strains revealed that the cell growth of the N-terminal truncation mutants was significantly enhanced compared to the control after IPTG induction. Among them, the growth rate of ∆N27 strain growth significantly, with OD_600_ 3.1 after 10 h of induction, which was 6.2 times higher than the control strain, effectively alleviating the growth inhibition from full length PlaS (Fig. 1E). Subsequently, SDS-PAGE analysis of different lengths of PlaS showed that N-terminal truncation not only promoted host growth but also significantly enhanced the solubility of the protein (Fig. 1F, Figure S1). These results demonstrate that N-terminal optimization is an effective means to promote the solubility of the protein. By truncating the N-terminal 27 amino acids of PlaS, the growth inhibition on the host cells was eliminated, leading to in-creased cell biomass and enhanced protein solubility of the protein.

### N-terminal truncation of PlaS enhances PlaA1 enzymatic activity

**Fig. 2:**
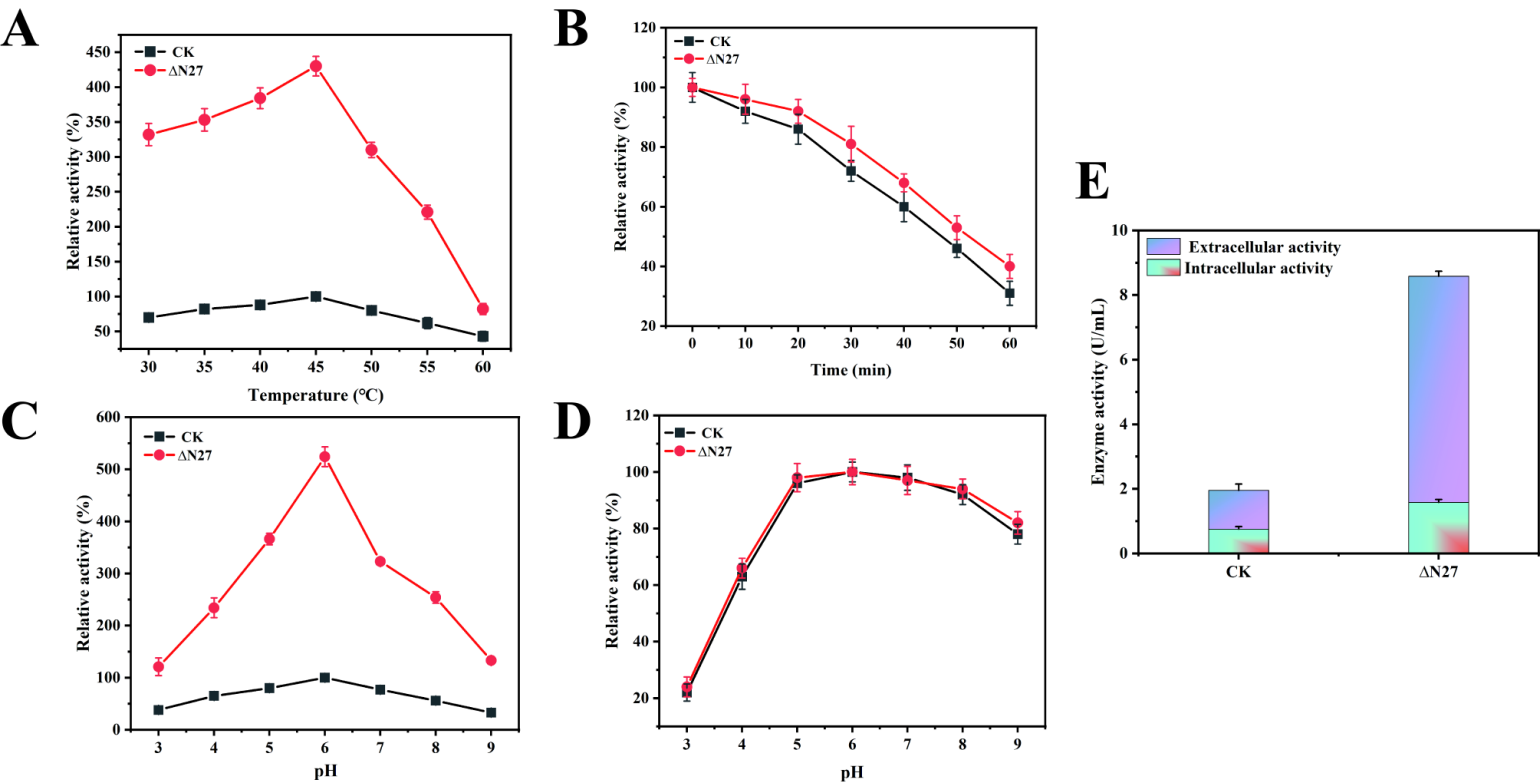
Enzymatic Characterization of PlaS and ∆N27 PlaS. (**A**) Enzyme activity of PlaA1 measured at different temperature, showing the optimal temperature for enzymatic activity. (**B**) Thermal stability of PlaA1-PlaS and ∆N27 PlaS, illustrating the residual enzyme activity after incubation at 45 °C for 60 min. (**C**) Enzyme activity of PlaA1-PlaS and ∆N27 PlaS at different pH values, highlighting the enhanced activity of ∆N27 PlaS. (**D**) pH stability of PlaA1-∆N27 PlaS, presenting the residual enzyme activity after incubation at different pH levels for 60 min. (**E**) Intracellular and extracellular enzymatic activity of PlaA1-PlaS and PlaA1-∆N27 PlaS

Although the N-terminal truncation of PlaS has been shown to enhance its expression level, further investigation is required to understand its impact on the enzymatic activity of phospholipase A1 (PlaA1). Therefore, we constructed recombinant strains PlaA1-PlaS and PlaA1-∆N27 PlaS using the expression vector pET28a in *E. coli* BL21 (Figure S2). By measuring the enzymatic properties of PlaA1 in these recombinant strains, we determined the enzyme activity of PlaA1-PlaS as the reference, set to 100%. Figure 2A shows the enzyme activity of PlaA1 at different temperatures. The optimal reaction temperature of PlaA1 was 45 °C, and the relatively high activity were observed between 35 °C and 50 °C. However, enzyme activity rapidly decreased when the temperature exceeded 55 °C. Furthermore, the stability of PlaA1-PlaS and PlaA1-∆N27 PlaS were investigated at different temperatures, we found that PlaA1-∆N27 PlaS maintained over 50% residual enzyme activity after incubation at 45 °C for 50 min, exhibiting improved thermal stability and half-life compared to PlaA1-PlaS (Fig. 2B).

Furthermore, we assessed the enzyme activity of PlaA1-PlaS and PlaA1-∆N27 PlaS at different pH values. Figure 2C and E depict the results, with the highest enzyme activity of PlaA1-PlaS set as 100%. The optimal reaction pH was 6.0 for PlaA1, while the intracellular and extracellular enzyme activities of PlaA1-∆N27 PlaS at this pH were 7 U/mL and 1.58 U/mL, respectively, showing 5.83-fold and 2.11-fold compared to PlaA1-PlaS. Also, PlaA1-∆N27 PlaS exhibited similar optimal pH and pH stability to PlaA1-PlaS, showing higher enzyme activity in alkaline conditions and lower activity in acidic conditions. The influence of pH on the stability of PlaA1-∆N27 PlaS was also investigated, and the residual enzyme activity was maintained above 80% after incubation at pH 5.0–9.0 for 60 min, showing great stability within this pH range (Fig. 2D).

Understanding the molecular basis of enzyme-substrate interactions is crucial for optimizing enzyme performance. Previous studies have demonstrated the benefits of N-terminal truncation of PlaS in enhancing protein expression and host cell growth. Here, we employed enzyme kinetics parameters to explore the impact of N-terminal truncation on the PlaA1 activity. Enzyme kinetics parameters were performed to investigate the enzymatic properties of PlaA1 in the truncated strain PlaA1-∆N27 PlaS. The determined kinetic parameters reveal a 27.1% decrease in km value and a 3.16-fold increase in catalytic efficiency (kcat/km) compared to PlaA1-PlaS (Table 1), the kcat value was also changed from 411.4032 to 946.6477. These findings suggest that N-terminal truncation of PlaS likely enhances the substrate affinity of PlaA1, leading to higher catalytic efficiency and enzymatic activity. These results further demonstrated that truncating the N-terminal 27 amino acids not only alleviates the growth inhibition on host cells but also significantly promotes the enzymatic activity of PlaA1.

**Table 1:** Kinetic parameters of PlaA1 in PlaA1-PlaS and PlaA1-∆N27 PlaS

Enzyme	km/(mg/mL)	V_max_/(U/(mg·min))	(kcat/ km)/(mL/(mg·s))
PlaA1-PlaS	0.3798 ± 0.0022	23.45 ± 1.21	1083.21 ± 87.34
PlaA1-∆N27 PlaS	0.2768 ± 0.0038	34.60 ± 0.68	3419.97 ± 58.72

### Intracellular investigation of the interaction between PlaS and PlaA1 through yeast two-hybrid assay

**Fig. 3:**
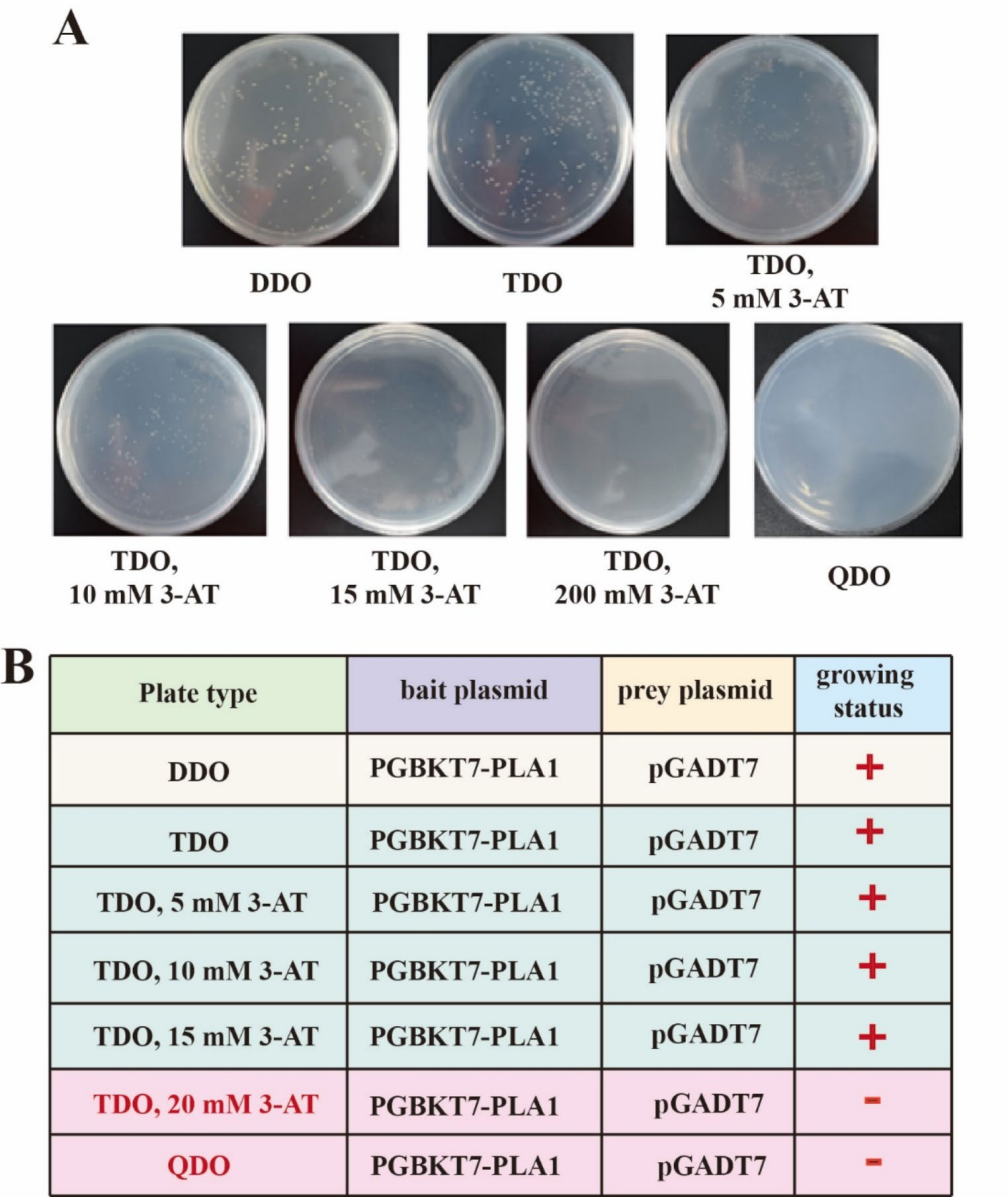
Detection of toxicity and self-activation of bait plasmids. (**A**) Interaction between PGBKT7-PLA1 and pGADT7 in yeast two-hybrid system on different plates. (**B**) Results of toxicity and self-activation of bait plasmids

Protein-protein interactions play a crucial role in cellular processes, and understanding these interactions is fundamental for elucidating biological mechanisms (Wang et al. 2023). Previous studies have shown that PlaS enhances the expression of PlaA1 and promotes its enzymatic activity. In this study, we employed yeast two-hybrid assays to investigate the potential interaction between PlaS or its truncated form, ∆N27 PlaS, and PlaA1. Firstly, the constructed bait plasmid pGBKT7-PlaA1 and empty prey plasmid pGADT7 were transformed into the yeast strain Y2HGold, toxicity and autoactivation tests were performed. The results, as shown in the Fig. 3A, indicate that both the empty prey plasmid pGBKT7 and the recombinant bait plasmid pGBKT7-PlaA1 allowed normal growth on DDO plates, demonstrating successful transformation into the host strain without toxicity. However, the Y2H yeast strain coated on the TDO plate could grow, indicating that the bait plasmid PGBKT7-PLA + PGADT7 activated the His3 reporter gene. Therefore, it is necessary to use different concentrations of 3-AT for background inhibition, and finally 20 mM of 3-AT can inhibit the expression of His3 reporter gene. Moreover, the Y2H yeast strain failed to grow on QDO plates, indicating the absence of autoactivation. These results suggest that the bait protein PlaA1 is non-toxic to yeast Y2HGold and does not activate the expression of ADE reporter genes, making it suitable for subsequent identification of interactions between PlaA1 and PlaS or ∆N27 PlaS. In summary, the agar plates of TDO 20mM 3-AT and QDO can be used for mutual verification in the following experiment (Fig. 3B).

**Fig. 4:**
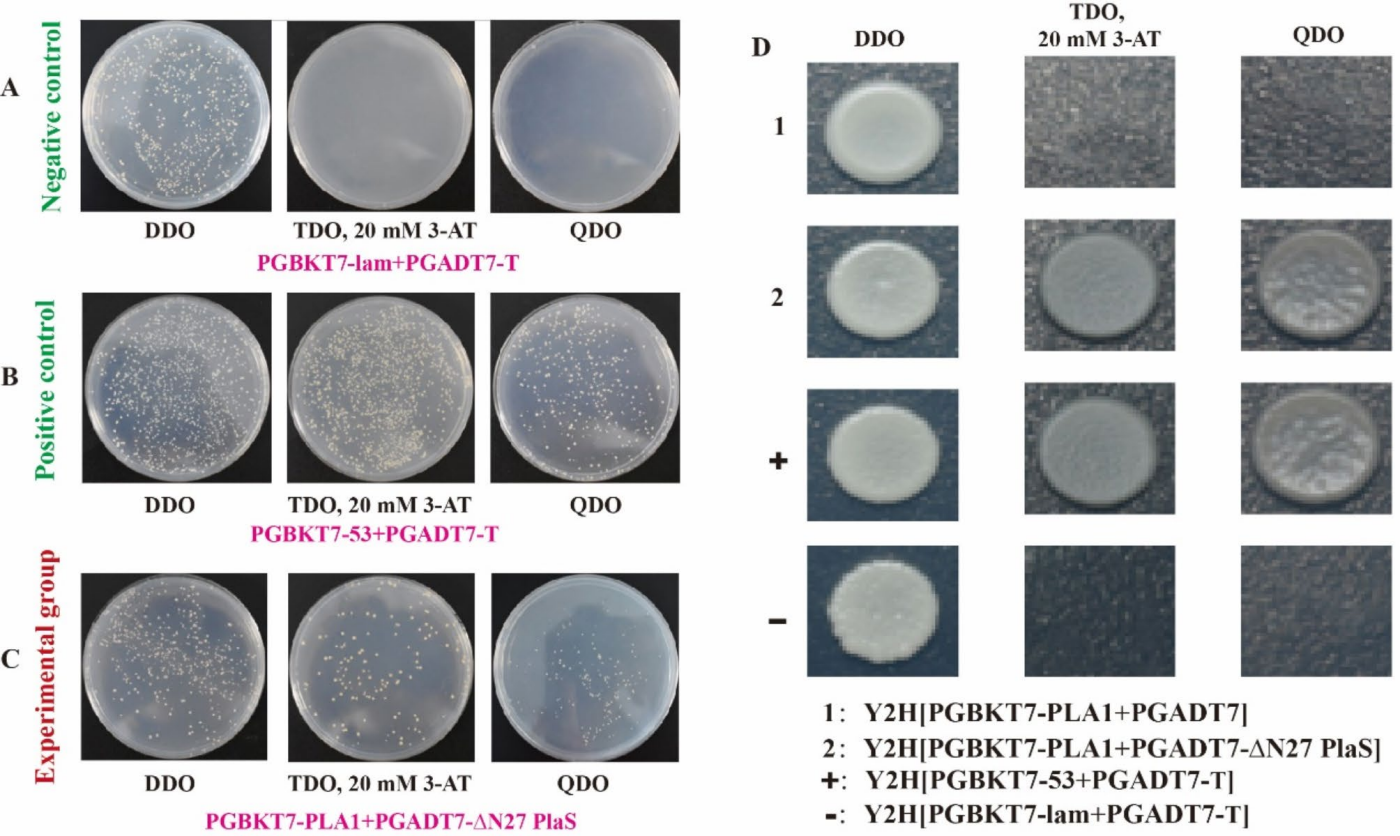
Yeast two-hybrid assay reveals the interaction between ∆N27 PlaS and PlaA1. (**A**-**C**) Co-transformation of prey plasmid and bait plasmid. (**D**) Spot board verification. (1:Y2H[PGBKT7-PLA1 + PGADT7]; 2: Y2H[PGBKT7-PLA1 + PGADT7 –S27]; +: Y2H[pGBKT7-53 + pGADT7-T];-: Y2H[pGBKT7-lam + pGADT7-T])

Subsequently, the successfully constructed prey plasmids pGADT7-∆N27 PlaS were co-transformed with the bait plasmid pGBKT7-PlaA1 into the yeast strain Y2HGold, and the yeast two-hybrid system was employed to investigate the potential interactions between ∆N27 PlaS and PlaA1, and negative control and positive control were set at the same time. As depicted in the Fig. 4A-B, the results of the control group were in line with expectations, indicating that the system could be used for two-hybrid assay verification. In the experimental group (Fig. 4C), the co-transformation results of the prey plasmids pGADT7-∆N27 PlaS with the bait plasmid pGBKT7-PlaA1 were consistent with the positive control, exhibiting growth on TDO 20mM 3-AT and QDO plates, indicating that the expression of His3 and ADE2 reporter genes is activated.

And also, the result of spot board verification was also consistent with those described above (Fig. 4D). These results suggest that both ∆N27 PlaS possess the ability to interact with PlaA1, and the expression of PlaS enhances the enzymatic activity of PlaA1.

### Biacore analysis reveals the interaction between ∆N27 PlaS and PlaA1 in vitro

**Fig. 5:**
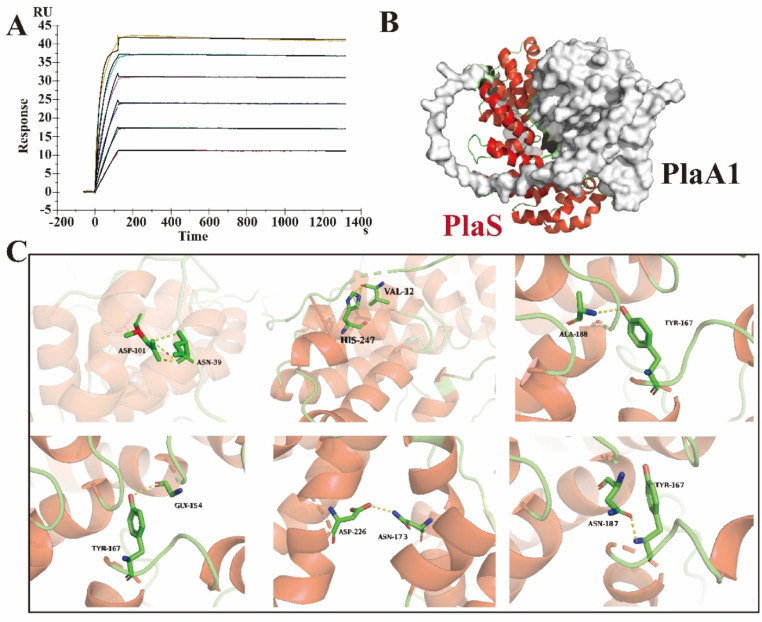
The interaction between ∆N27 PlaS and PlaA1 in vitro. (**A**) Biacore analysis revealed specific binding between ∆N27 PlaS and PlaA1, where the ∆N27 PlaS protein was diluted to concentrations of 0, 0.156, 0.3125, 0.625, 1.25, 2.5, and 5 µM. (**B**) Molecular docking model of the PlaA1-∆N27 PlaS protein complex. (**C**) Analysis of hydrogen bond interactions in the PlaA1-∆N27 PlaS protein complex

To further explore the interaction mechanism between ∆N27 PlaS and PlaA1 in vitro, purified PlaA1 and ∆N27 PlaS enzymes were freeze-dried to obtain high-purity protein lyophilized powders. PlaA1 protein was diluted to a concentration of 20 µg/mL, while ∆N27 PlaS protein was diluted to concentrations of 0, 0.156, 0.3125, 0.625, 1.25, 2.5, and 5 µM. The binding affinity between different concentrations of ∆N27 PlaS and PlaA1 was assessed using Biacore T200 (GE Healthcare). The results demonstrated (Fig. 5A) a specific and concentration-dependent binding between PlaA1 and ∆N27 PlaS, with a binding rate constant (Ka) of 1479 Ms^− 1^, dissociation rate constant (Kd) of 1.255E-5 s^− 1^, and equilibrium dissociation constant (KD) of 8.486E-9 M (Table 2). The experimental results indicate that the phospholipase PlaA1 and the assisting protein ∆N27 PlaS exhibit specific binding.

**Table 2:** Biacore analysis of the interaction between ∆N27 PlaS and PlaA1

Ligand	Analyte	Ka (1/Ms)	Kd (1/s)	KD (M)
PlaA1	∆N27 PlaS	1479	1.255E-5	8.486E-9

To further investigate the interaction mechanism between ∆N27 PlaS and PlaA1, homology models of PlaA1 and ∆N27 PlaS were constructed, and molecular docking was performed using the ZDOCK program in Discovery Studio 2016, resulting in the PlaA1-∆N27 PlaS protein complex (Fig. 5B). Analysis of hydrogen bond interactions revealed that ASP101, HIS247, ALA188, GLY154, ASP226, and ASN187 in ∆N27 PlaS form hydrogen bonds with ASN39, VAL12, TYR167, ASN173, and TYR167 in PlaA1, respectively. Among them, TYR167 in PlaA1 interacts with ALA188, GLY154, and ASN187 through hydrogen bonds simultaneously (Fig. 5C). Therefore, these results suggested that the ALA188, GLY154, and ASN187-related residues in ΔN27 PlaS may be involved in binding with PlaA1, providing a research direction for further enhancing the enzymatic activity of PlaA1 through site-directed mutagenesis.

### Boost the expression of phospholipase A1 in *Escherichia coli* by orthogonal test

**Fig. 6:**
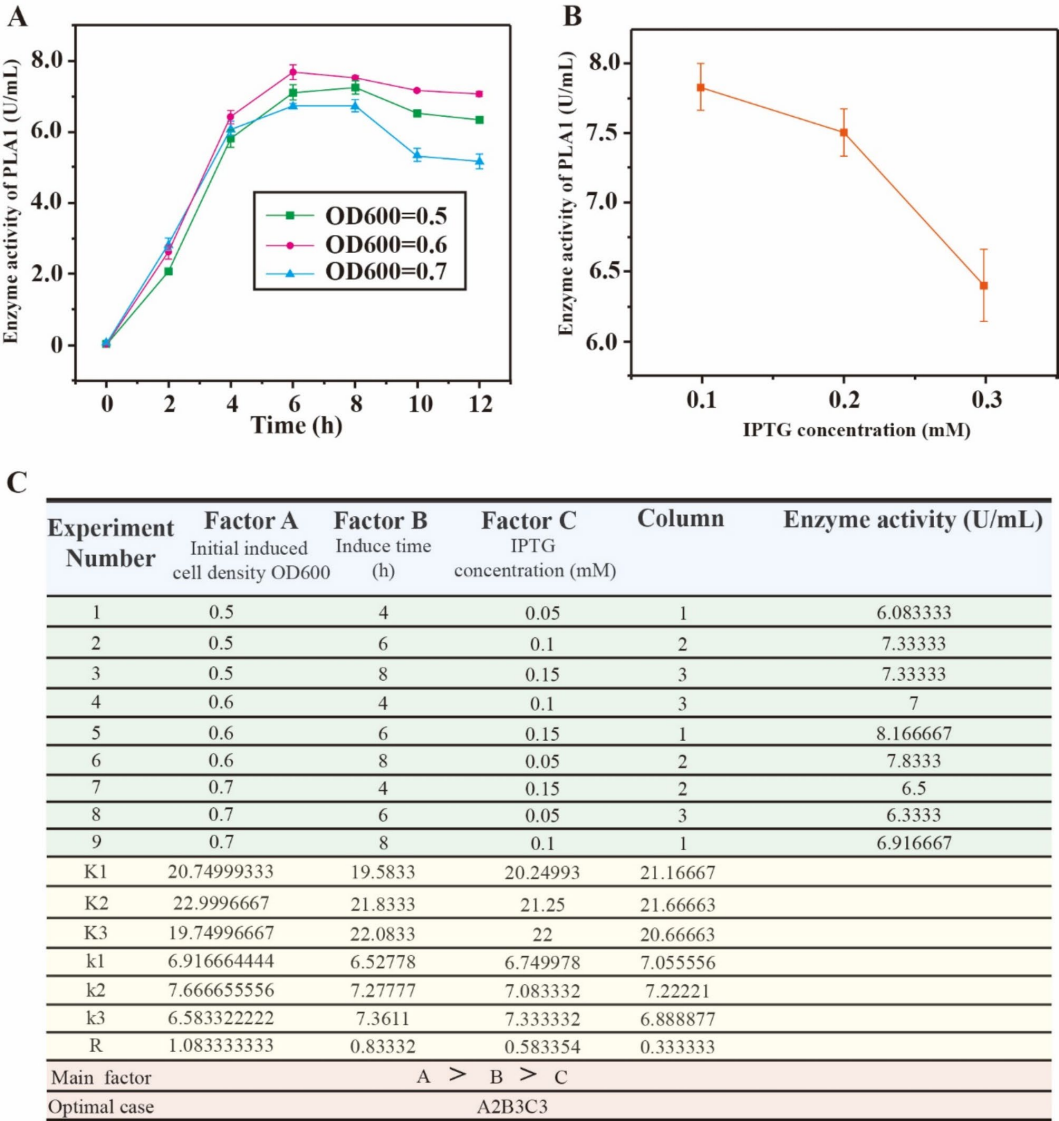
Optimization of inducing PLA1 expression conditions. (**A**) Time curve of the effect of different initial induced cell OD value on enzyme activity. (**B**) Enzyme activity under different IPTG concentrations. (**C**) Orthogonal experiment results of phospholipase A1 activity of co-expressed strain BL21/pET28a-PlaA1-∆N27 PlaS

In order to further enhance the enzyme activity, the effects of initially induced cell density OD600, induced time and IPTG concentration on the extracellular phospholipase A1 activity of co-expressing strain BL21/pET28a-PlaA1-∆N27 PlaS were investigated. Firstly, the influence of OD600 value of different initially induced cells on enzyme activity was studied (Fig. 6A), and it was found that OD600 was 0.6 and the enzyme activity reached the highest level after 6 h of induction. On this basis, the effect of IPTG concentration on enzyme activity was explored (Fig. 6B), and the results showed that enzyme activity was highest when IPTG concentration was 0.1mM.

Based on the above results, orthogonal experiments with 3 factors and 3 levels were designed (Fig. 6C). From the results of orthogonal experiments, it is concluded that the optimal combination is A2B3C3. Since the experimental group did not cover this scheme, the enzyme activity of the optimal scheme obtained by orthogonal was re-determined, and the final result was that the enzyme activity was 8.41666 U/mL at the combination A2B3C3, and the optimal condition for inducing PLA1 expression was to add 0.15mmol/L IPTG for 8 h when the strain BL21/pET28a-PlaA1-∆N27 PlaS grew to an OD600 of 0.6.

## Conclusions

In summary, our research elucidates the role of the phospholipase-assisting protein PlaS in enhancing the expression and enzymatic activity of PlaA1. Truncation of the N-terminal region of PlaS effectively overcame its inhibitory effect on host cells, and increased protein solubility of the protein. The yeast two-hybrid assay confirmed the interaction between PlaA1 and PlaS, ∆N27 PlaS, demonstrating their binding capabilities. In vitro studies using Biacore analysis revealed a concentration-dependent and specific binding between PlaA1 and ∆N27 PlaS with high affinity. Molecular docking analysis provided insights into the hydrogen bond interactions between ∆N27 PlaS and PlaA1, identifying key amino acid residues crucial for their binding. These findings suggest that PlaS could enhance its enzymatic activity. Finally, the enzyme activity of PLA1 reached a high level by optimizing the induced conditions. Overall, our study contributes to a better understanding of the interaction mechanism between PlaS and PlaA1 and provides a potential avenue for enhancing PlaA1 activity through protein engineering strategies.

### Electronic supplementary material

Below is the link to the electronic supplementary material.

Supplementary Material 1

Supplementary Material 2

## Data Availability

All data that support the findings of this study are included within this paper/supplementary files.
