# Supplementary material for: N-terminal truncated phospholipase A1 accessory protein PlaS from Serratia marcescens alleviates inhibitory on host cell growth and enhances PlaA1 enzymatic activity
Source: Bioresour Bioprocess. 2024 Jun 25;11(1):61. doi: 10.1186/s40643-024-00777-1 (PMC11199421; doi:10.1186/s40643-024-00777-1)
Supplement: Supplementary file 1 — Supplementary Material 1 [file 40643_2024_777_MOESM1_ESM.docx]

**Supporting Information**

**N-terminal truncated Phospholipase A1 accessory protein PlaS from *Serratia marcescens*** **alleviates inhibitory on host cell growth and enhances PlaA1 enzymatic activity**

Mengkai Hu ^a,1^, Jun Liu ^a,1^, Yufei Gan ^a^, Hao Zhu^a^, Rumeng Han^a^, Kun Liu ^a^, Yan Liu^a^, Ming Zhao ^a^, Xiangfei Li ^a,^* and Zhenglian Xue ^a,^*

^a^ Engineering Laboratory for Industrial Microbiology Molecular Beeding of Anhui Province, College of Biologic & Food Engineering, Anhui Polytechnic University, 8 Middle Beijing Road, Wuhu 241000, China

^1^ Mengkai Hu and Jun Liu have contributed equally to this work.

***** Corresponding authors:

Prof. Zhenglian Xue, E-mail: xzlahpu@163.com

PhD. Xiangfei Li, E-mail: [xiangfei@ahpu.edu.cn](mailto:xiangfei@ahpu.edu.cn)

Email address:

[mengkaihu0725@163.com](mailto:mengkaihu0725@163.com) (Mengkai Hu); [2442996761@qq.com](mailto:2442996761@qq.com) (Jun Liu)

[2551864178@qq.com](mailto:2551864178@qq.com) (Yufei Gan); [zhuhao0722@126.com](mailto:zhuhao0722@126.com) (Hao Zhu)

[17851313515@163.com](mailto:17851313515@163.com) (Rumeng Han); [liuk@mail.ahpu.edu.cn](mailto:liuk@mail.ahpu.edu.cn) (Kun Liu)

[1766187455@qq.com](mailto:1766187455@qq.com) (Yan Liu) ; [zmahpu@163.com](mailto:zmahpu@163.com) (Ming Zhao)

[xiangfei@ahpu.edu.cn](mailto:xiangfei@ahpu.edu.cn) (Xiangfei Li); [xzlahpu@163.com](mailto:xzlahpu@163.com) (Zhenglian Xue)

**Table S1.** Strains and plasmids used in this study

|  | Characteristics | Source |
| --- | --- | --- |
| **Strains** |  |  |
| *E. coli* BL21 | Host for recombinant protein production | Novagen |
| BL21/pET28a | *E*. *coli* BL21 derivative harboring pET28a | The lab |
| BL21/pET28a-PlaA1 | *E*. *coli* BL21 derivative harboring pET28a-PlaA1 | The lab |
| BL21/pET28a-PlaS | *E*. *coli* BL21 derivative harboring pET28a-PlaS | This study |
| BL21/pET28a-∆N27 PlaS | *E*. *coli* BL21 derivative harboring pET28a-∆N27 PlaS | This study |
| BL21/pET28a-PlaA1-PlaS | *E*. *coli* BL21 derivative harboring pET28a-PlaA1-PlaS | This study |
| BL21/pET28a-PlaA1-∆N27 PlaS | *E*. *coli* BL21 derivative harboring pET28a-PlaA1-∆N27 PlaS | This study |
| Y2HGold (Y2H) | Yeast two-hybrid experimental strain suitable for GAL4 system, MATa type. | Clontech |
| Y2H/pGBKT7-PlaA1 | Y2HGold derivative harboring pGBKT7-PlaA1 | This study |
| Y2H/pGADT7-PlaS | Y2HGold derivative harboring pGADT7-PlaS | This study |
| Y2H/pGADT7-∆N27 PlaS | Y2HGold derivative harboring pGADT7-∆N27 PlaS | This study |
| **Plasmids** |  |  |
| pET28a | Expression vector for the expression of target protein | Novagen |
| pET28a-PlaA1 | Derived from pET28a, for induced expression of *plaA1* | This study |
| pET28a-PlaS | Derived from pET28a, for induced expression of *plaS* | This study |
| pET28a-∆N27 PlaS | Derived from pET28a, for the expression of ∆N27 *plaS* | This study |
| pET28a-PlaA1-PlaS | Derived from pET28a, for the co-expression of *plaA1*-*plaS* | This study |
| pET28a-PlaA1-∆N27 PlaS | Derived from pET28a, for the co-expression of *plaA1*-∆N27 *plaS* | This study |
| pGBKT7 | Yeast two-hybrid "bait" vector for expressing proteins fused to the GAL4 DNA-binding domain (DNA-BD). | Clontech |
| pGADT7 | Yeast two-hybrid " prey " vector for expressing proteins of interest fused to the GAL4 activation domain (AD). | Clontech |
| pGBKT7-53 | DNA-BD Control Vector, a positive control plasmid that encodes a fusion of the murine P53 protein and the GAL4 DNA-BD. | Clontech |
| pGBKT7-lam | DNA-BD Control Vector, a negative control plasmid that encodes a fusion of the human lamin C protein and the GAL4 DNA-BD. | Clontech |
| pGADT7-T | AD control plasmid, a positive control plasmid that encodes a fusion of the SV40 large T antigen protein and the GAL4 AD. | Clontech |
| pGBKT7-PlaA1 | Derived from pGBKT7, for the co-expression of *plaA1* | This study |
| pGADT7-PlaS | Derived from pGADT7, for the co-expression of *plaS* | This study |
| pGADT7-∆N27 PlaS | Derived from pGADT7, for the co-expression of ∆N27 *plaS* | This study |

**Table S2.** Primers used in this study

| Primer name | Sequences |
| --- | --- |
| plaS F | AGCAAATGGGTCGCGGATCCATGCCTGAAGGGCGTCG |
| plaS R | TGTCGACGGAGCTCGAATTCTTACTGCTGCGCGTAGTGC |
| ∆N27 PlaS F | AGCAAATGGGTCGCGGATCCATGGCTAAGGAGCAACAGATGGGG |
| ∆N27 PlaS R | TGTCGACGGAGCTCGAATTCTTACTGCTGCGCGTAGTGC |
| pGBKT7-PlaA1 F | CTCGGAATTCATGGGCAGTATGCCTT |
| pGBKT7-PlaA1 R | CTCGGGATCCTCAGGCATTGGCCTT |
| pGADT7-PlaS F | CTCGGGATCCATGTACCTGAAGGGCG |
| pGADT7-∆N27 PlaS F | GCTCATATGGCCATGGAGGCCAGTGAATTCGAGATTT  CACCGTTTGACGG |
| pGADT7-∆N27 PlaS R | ATTCATCTGCAGCTCGAGCTCGATGGATCCCAGACTGCTGCGCGTAGTGCG |

**Table S3.** Design of three factors and three levels

| Experiment  number | Factor A | Factor B | Factor C |
| --- | --- | --- | --- |
|  | Initial induced  cell density OD600 | Induced time  (h) | IPTG  Concentration (mM) |
| 1 | 0.5 | 4 | 0.05 |
| 2 | 0.5 | 6 | 0.1 |
| 3 | 0.5 | 8 | 0.15 |
| 4 | 0.6 | 4 | 0.1 |
| 5 | 0.6 | 6 | 0.15 |
| 6 | 0.6 | 8 | 0.05 |
| 7 | 0.7 | 4 | 0.15 |
| 8 | 0.7 | 6 | 0.05 |
| 9 | 0.7 | 8 | 0.1 |

**Table S4. ANOVA analysis**

| Sources | Sum of squares | dF | Mean square | F-Values | P-Values | Significance |
| --- | --- | --- | --- | --- | --- | --- |
| A | 3.347500222 | 2 | 1.67375 | 20.08497 | 0.047427 | significant |
| B | 1.263888889 | 2 | 0.881944 | 10.58332 | 0.086331 |  |
| C | 0.514500222 | 2 | 0.40725 | 4.886995 | 0.169866 |  |
| residuals | 0.166666889 | 2 | 0.083333 |  |  | not significant |

**Figure S1**


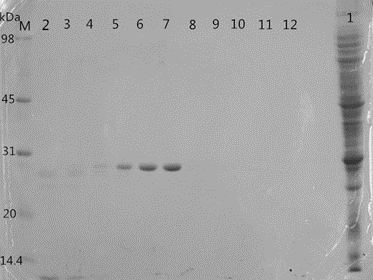
The full uncropped Gels and Blots image(s) corresponds to Figure 1F

**Figure S2** SDS-PAGE analysis of PlaA1 purification under the assisting protein PlaS and ∆N27 PlaS


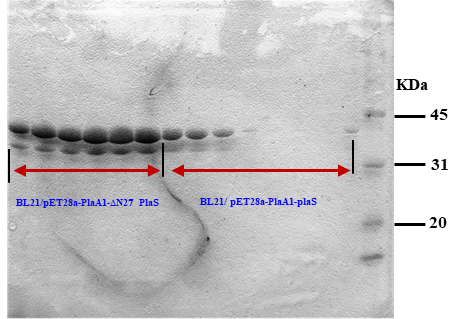


**
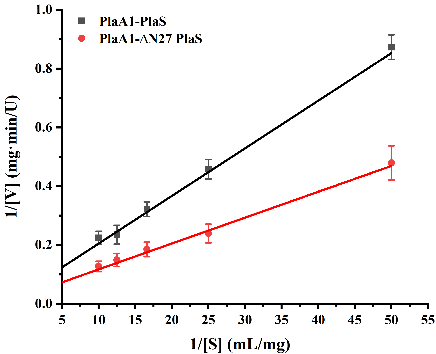
Figure S3 Lineweaver-Burk plot**
