# Supplementary figures and images for: N-terminal truncated phospholipase A1 accessory protein PlaS from Serratia marcescens alleviates inhibitory on host cell growth and enhances PlaA1 enzymatic activity
Source: Bioresour Bioprocess. 2024 Jun 25;11(1):61. doi: 10.1186/s40643-024-00777-1 (PMC11199421; doi:10.1186/s40643-024-00777-1)

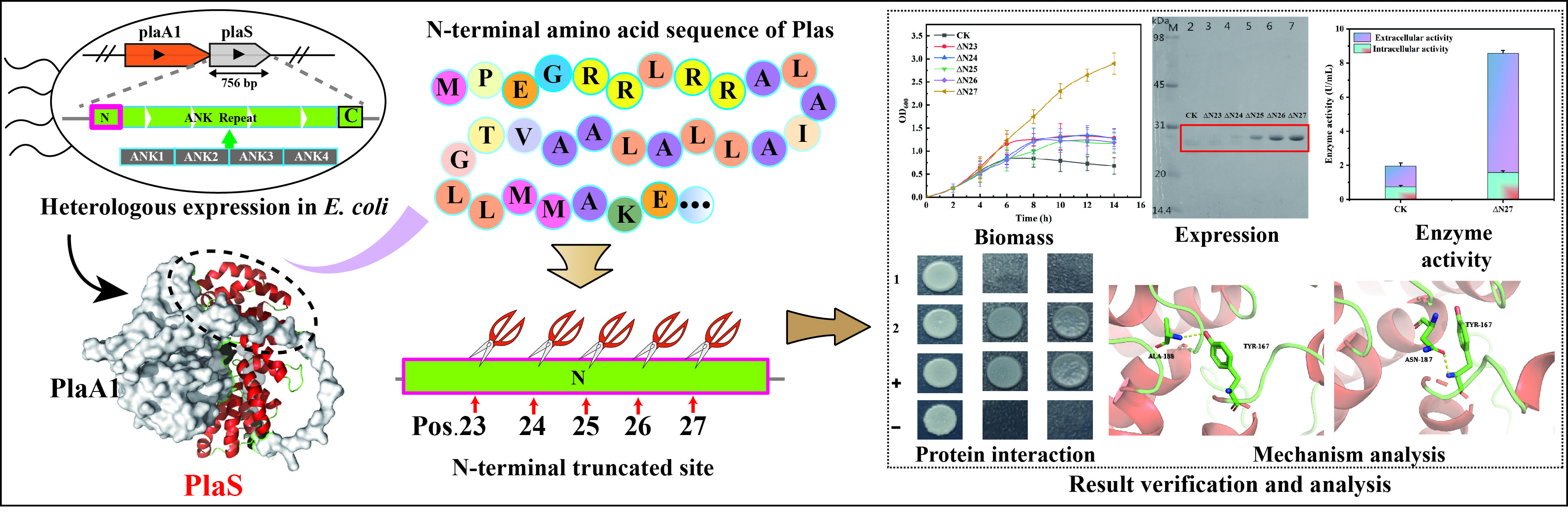

Supplement: Supplementary file 2 — Supplementary Material 2 [file 40643_2024_777_MOESM2_ESM.jpg]
